# Supplementary material for: Empirical methods for controlling false positives and estimating confidence in ChIP-Seq peaks
Source: BMC Bioinformatics. 2008 Dec 5;9:523. doi: 10.1186/1471-2105-9-523 (PMC2628906; doi:10.1186/1471-2105-9-523)
Supplement: Additional File 1 — A variety of html documents from the USeq web site detailing the available applications, their best usage, output file type descriptions, command line menus, etc. [file 1471-2105-9-523-S1.zip › USeqUserGuides/JavaDoc.zip!/JavaDoc/util/apps/package-frame.html]

util.apps


util.apps

Classes 
  
ArrayPrinter
  
Correlate
  
Delme2
  
FileJoiner
  
FileSplitter
  
FileTrimmer
  
FilterAgilentProbeFile
  
Flash
  
GeneCentricCNVParser
  
GeneNameCounter
  
HouseBean
  
IntersectLists
  
JQSub
  
MakeBioTools
  
MergeColumns
  
ParseMLSHTML
  
ParserForGff
  
PrintSelectColumns
  
RenameRWSResults
  
ScatterDrawFrame
  
ScatterDrawPanel
  
ScatterPlot
  
ScoreChromosomes
  
ScoreSequences
  
SmoothData
  
StatBarFiles
  
SubtractOneFromEachColumn
  
TASSignalChromFileSplitter
  
TextParser
  
WindowScoreSmoother
  
ZipAndDelete
